# The Effect of Radiation Emitted by Cell Phone on The Gelatinolytic Activity of Matrix Metalloproteinase-2 and -9 of Mouse Pre-Antral Follicles during *In Vitro* Culture

**DOI:** 10.22074/cellj.2020.6548

**Published:** 2019-09-08

**Authors:** Fariba Azimipour, Saeed Zavareh, Taghi Lashkarbolouki

**Affiliations:** 1 School of Biology, Damghan University, Damghan, Iran; 2 Institute of Biological Sciences, Damghan University, Damghan, Iran

**Keywords:** Cell Phone, Gelatinase, Ovarian Follicles, Radiation

## Abstract

**Objective:**

The unfavorable effects of electromagnetic radiation (EMR) emitted by the cell phone on reproduction health are controversial. Metalloproteinases play a vital role in ovarian follicle development. This study was designed to investigate the effects of exposure to the cell phone on the gelatinolytic activity of *in vitro* cultured mouse pre-antral follicle.

**Materials and Methods:**

In this experimental study, pre-antral follicles were isolated from ovaries of immature mice (n=16) and cultured with or without exposure to the cell phone in talking mode for 60 minutes. The gelatinolytic activity was evaluated through the zymography method, as well as the gene expression of matrix metalloproteinases (MMPs) namely MMP-2 and -9 and tissue inhibitors of metalloproteinases (TIMPs) namely, *TIMP-1* and -2 by the real-time polymerase chain reaction (PCR) method. Also, in parallel, the development of pre-antral follicles was assessed.

**Results:**

The maturation parameters of the cell phone-exposed pre-antral follicles were significantly lower compared with the control group (P<0.05). The gelatinolytic activity was significantly decreased in the cell phone-exposed pre- antral follicles compared with the control group (P<0.05). The relative mRNA expression of the MMP-2 gene was significantly (P<0.05) increased in the cell phone-exposed pre-antral follicles whereas the expression rate of the *MMP-9* gene was considerably (P<0.05) reduced when compared with the control group. Conversely, the relative expression of the *TIMP-1* was markedly (P<0.05) increased in the cell phone-exposed pre-antral follicles while the expression of the *TIMP-2* was (P<0.05) significantly diminished in comparison with the control group.

**Conclusion:**

Exposure to the cell phone alters the growth and maturation rate of murine ovarian follicle through the changing in the expression of the MMP-2 and -9 genes, as well as the gelatinolytic activity.

## Introduction

Nowadays, the usage of mobile phone and exposure to its electromagnetic radiation (EMR) raised some concerns about the dangerous effects on health. EMR affect the cells and organs through thermal and non-thermal mechanisms (1). However, there are some controversial reports, indicating EMR may increase the free radical production, mitochondrial dysfunction, heat shock proteins, apoptosis, and DNA damage (2-5). Besides, EMR may target the plasma membrane and disturb various enzymatic activities and the receptor functions (6). It is also shown that exposure to EMR increases the NADH oxidase activity of the plasma membrane which subsequently changes the activation of matrix metalloproteinase (MMPs) (7) b¿. The interaction of MMPs and tissue inhibitor of metalloproteinases (TIMPs) regulates the extracellular matrix (ECM) remodeling in many physiological processes (8) which could subsequently maintain cellular homeostasis (9). 

The development of ovarian follicle is accompanied by immense cellular turnover and remodeling of ovarian tissue. The essential part of this remodeling is the modification of the ECM constituents and provision of the structural support for the follicle formation and maturation. Among MMPs, gelatinase A (*MMP-2*) and B (*MMP-9*) play significant roles in follicle development and ovulation (10). Furthermore, *TIMP-1* and *TIMP-2* bind to *MMP-9* and *MMP-2*, respectively, and regulate their activations. *TIMP-1* regulates the rate of proteolysis within the granulosa cells through the ovulatory process. By contrast, *TIMP-2*, in the thecal cells, promotes the proteolysis process via the localization of pro-gelatinase A on the cell surface which may be used as biomarkers of the normal follicle development (10, 11).

There is limited information about the effect of EMR, emitted from the cell phone on female reproductive tissue. However, no report is available about the effect of EMR on different aspects of folliculogenesis. The present study aimed to investigate whether exposure to cell phone radiation affects the gelatinolytic activity of *MMP-2* and MMP-9, as well as the mRNA expression of *MMP-2* and -9 along with their inhibitors *TIMP-1* and *-2* in mouse pre-antral follicles during *in vitro* culture.

## Materials and Methods

### Reagents

Unless otherwise specified, all reagents and chemical were obtained from Sigma Aldrich (UK). Water used for the preparation of the culture medium was purified by the Milli-Q system. 

### Animals

In this experimental study, all experiments were conducted using female Naval Medical Research Institute (NMRI) mice which were obtained from the Razi Vaccine and Serum Research Institute. Female offspring with age range of 14-16 days was used for all experiments. Animal experiments were performed based on the ethical principles of the Declaration of Helsinki as revised in Tokyo 2004 and was approved by the Ethics Committee of Damghan University. The animals were kept and bred under the standard conditions with a circadian rhythm of 12 hours of light/12 hours of darkness and at an ambient temperature of 22 ± 2°C with adequate food and water. 

### Experimental design

Mice were sacrificed through cervical dislocation, and their ovarian tissues were aseptically removed using scissors and forceps and put into 100 µl of the alpha-minimum essential medium (α-MEM) containing 25 mM HEPES, 70 µg/mL streptomycin, 100 IU/ml penicillin, 10% fetal bovine serum (FBS, Gibco, UK), and 2.2 g/l sodium bicarbonate. The preantral follicles with the diameter of 140-160 µm were mechanically isolated and selected based on the previously described criteria (intact with at least two to three granulosa cell layers and a centrally located oocyte) (12). The pre-antral follicles were randomly grouped into control (n=240) and cell phone-exposed groups (n=240). Exposed pre-antral follicles were kept in close to the commercial cell phone (Sony Ericsson K800) in the talking mode at 5 cm distance for 60 minutes. After that, the cell phone was removed, and pre-antral follicles were cultured for up to 12 days. The same protocols were applied for the control group except they were not exposed to the cell phone radiation. Some of the cultured pre-antral follicles were used for the assessment of the growth rate, and the remained cells were used for the molecular analyses. 

### *In vitro* maturation of pre-antral follicles 

Cultivation of pre-antral follicles were performed in which the cells were covered with embryo-tested mineral oil in 25-µL drops of the α-MEM medium containing 100 mU/ml recombinant human follicle stimulating hormone (rhFSH), 5% FBS, 20 ng/ml recombinant epidermal growth factor (rEGF), and 1% insulin-transferring-selenium (ITS), in an incubator at 37°C in 5% CO_2_ for 10 days, as previously described (12). The fresh maturation medium was replaced every two days. During the cultivation period, pre-antral follicles diameter was calculated under an inverted microscope at ×400 magnification by calculating the average of two perpendicular diameters with a precalibrated ocular micrometer in 2^nd^ and 4^th^ days of the culture period. The antrum cavity was defined as every lucent area between the granulosa cells and degenerated pre-antral follicles were detected with the darkness surrounding the cumulus cells and follicles without oocytes or denuded oocytes. In the 10^th^ days of the cultivation period, ovulation was induced by the addition of 1.5 IU/ml human chorionic gonadotropin (hCG, Choriomon, IBSA, Switzerland). After 2448 hours, oocytes were categorized based on the maturation status [germinal vesicle (GV), germinal vesicle breakdown (GVBD), and metaphase II oocytes (MII)]. 

### Gelatin zymography 

The gelatinase activity of MMP-2 and -9 was assessed using zymography on polyacrylamide gels containing gelatin, as described previously with some modifications (13). An equal amount of the culture medium was harvested during the culture period (days of 2, 4, 6, 8, 10, and 12). The medium was mixed and homogenized with an equal volume of non-reducing buffer containing 2% sodium dodecyl sulfate (SDS); Tris-HCl, (125 mM, pH=6.8), glycerol (10% v/v), and bromophenol blue (0.001% v/v). Afterward, the mixture was electrophoresed in 10% SDS page supplemented with gelatin (0.05% w/v). The gel was then washed twice in Triton X-100 (2%) for 20 minutes at room temperature and incubated in digestion buffer supplemented with Tris-HCl (50 mM), CaCl_2_ (2.5 mM) NaCl (200 mM), and ZnCl2 (1 mM) in pH=7.4 at 37°C overnight. Finally, the gel was stained with 0.5% Coomassie Brilliant Blue (Bio-Rad, Canada), and the destaining process was performed in glacial acetic acid (10% v/v) and methanol (30% v/v) dissolved in H2O for five hours at room temperate. The gelatinase activity was characterized based on unstained bands, and the quantification was performed using a computerized image analysis program (Image J) which, in turn, quantified the intensity and surface of unstained bands. 

### Reverse transcription-quantitative polymerase chain reaction

Real-time polymerase chain reaction (PCR) was performed to evaluate the relative mRNA expressions of the *MMP-2* and *-9* genes, as well as the *TIMP-1* and *-2* genes in pre-antral follicles during the cultivation period at the initial time and days of 6 and 12.

### RNA extraction

Total RNA extraction was carried out, as described previously on the basis of using acid guanidinium thiocyanate-phenol-chloroform (14, 15). In brief, pre-antral follicles (n=15 for each replicate) were placed in a 500 µl solution consisted of guanidine thiocyanate (4 M), sodium citrate (25 mM, pH=7.0), N-lauroylsarcosine (0.5% w/v), and 2-Mercaptoethanol (3.6 µl), and it was homogenized at room temperature. The homogenate was mixed with sodium acetate (50 µl), phenol (500 µl), and chloroform (200 µl) at 4°C and centrifuged at 12,000 g at 4°C for 20 minutes. Cold isopropanol was added to the resultant supernatants and placed at -20°C for 20 minutes and centrifuged (12,000 g) for 20 minutes. The resultant pellet was rinsed with 75% ethanol and diluted in RNase-free water. DNase was used to remove any DNA contamination. The RNA quality and quantity were evaluated using a density ratio of 28S to 18S rRNA bands and the measurement of its absorbance at A260 nm with the spectrophotometer. An A260 of 1.0 was considered 40 µg/mL of the extracted RNA. Also, the ratio of A260 to A280 nm was measured, and the samples with an A260 to A280 ratio of 1.8 to 2.0 were acceptable and used for reverse transcription. 

### Real-time polymerase chain reaction

The gene-specific primer sets were designed to span introns or cross exon/exon junctions, using the Oligo software version 7 (DBA Oligo, Inc., USA). All primer pairs were specific for the corresponding mRNAs and were tested for no amplification of genomic DNA. The contamination of genomic DNA in the sample was evaluated by performing a control reaction possessing no reverse transcribed RNA. Real- time PCR primer sequences and the thermal conditions are shown in Table 1, based on the MIQE (Minimum Information for Publication of Quantitative Real-Time RT-PCR Experiments) (16). The process of reverse transcription was accomplished by the synthesis of the first-strand cDNA using 1 µg of total RNA, Random Hexamer Primers (Fermentas, USA), Ribolock RNase Inhibitor, dNTP mix (Fermentas, USA), RevertAid M-Mul V reverse transcriptase (Fermentas, USA), 5X reaction buffer (Fermentas, USA), and RNase-free water according to the manufacturer’s instructions. The reaction was run in a thermocycler (Eppendorf, USA) with a thermal profile of 65°C for 5 minutes and one cycle of 42°C for 1 hour. Real-time RT-qPCR was performed on an ABI Step One machine (Applied Biosystems, ABI, USA) using the RT-PCR Kit (SYBR Green, Amplicon, Denmark). 

The thermal profile was adjusted to denaturation at 95°C for 15 minutes, followed by 40 cycles of denaturation at 95°C for 60 seconds, annealing and extension at 60°C for 60 seconds. The relative expression of the target genes was normalized against *GAPDH*. The 2^–ΔΔCt^ method was used to assess the relative gene expression for each gene. The specificity of real-time PCR was assessed through the melting curve analysis. 

### Statistical analysis

The SPSS software (version 24, Chicago, IL, USA) was used for the analysis of the data. Experiments were repeated at least four times. All data were expressed as the mean ± SD. Independent samples t test was applied for the determination of differences between groups. A P<0.05 was considered statistically significant. 

**Table 1:** Oligonucleotide primer sequences for real time polymerase chain reaction


Gene	Primer sequence (5ˊ-3ˊ)	(bp) Length	Product size

*MMP9*	F: CTGTCCAGACCAAGGGTACAG	20	247
	R: CATAGTGGGAGGTGCTGTCG	21	
*MMP2*	F: GAGAAGGACAAGTGGTCCGC	20	265
	R: CTGTTGTAGGAGGTGCCCTG	20	
*TIMP1*	F: GGGTGTGCACAGTGTTTCCC	22	202
	R: TTCAGTTTTTCCTGGGGGAAGG	20	
*TIMP2*	F: GCAGACGTAGTGATCAGAGCC	20	281
	R: TCCCAGGGCACAATGAAGTC	21	
*GAPDH*	F: TGACATCAAGAAGGTGGTGAAGC	22	203
	R: CCCTGTTGCTGTAGCCGTATTC 3	23	


## Results

### Effect of electromagnetic radiation on the maturation parameters of pre-antral follicles

**Fig.1:**
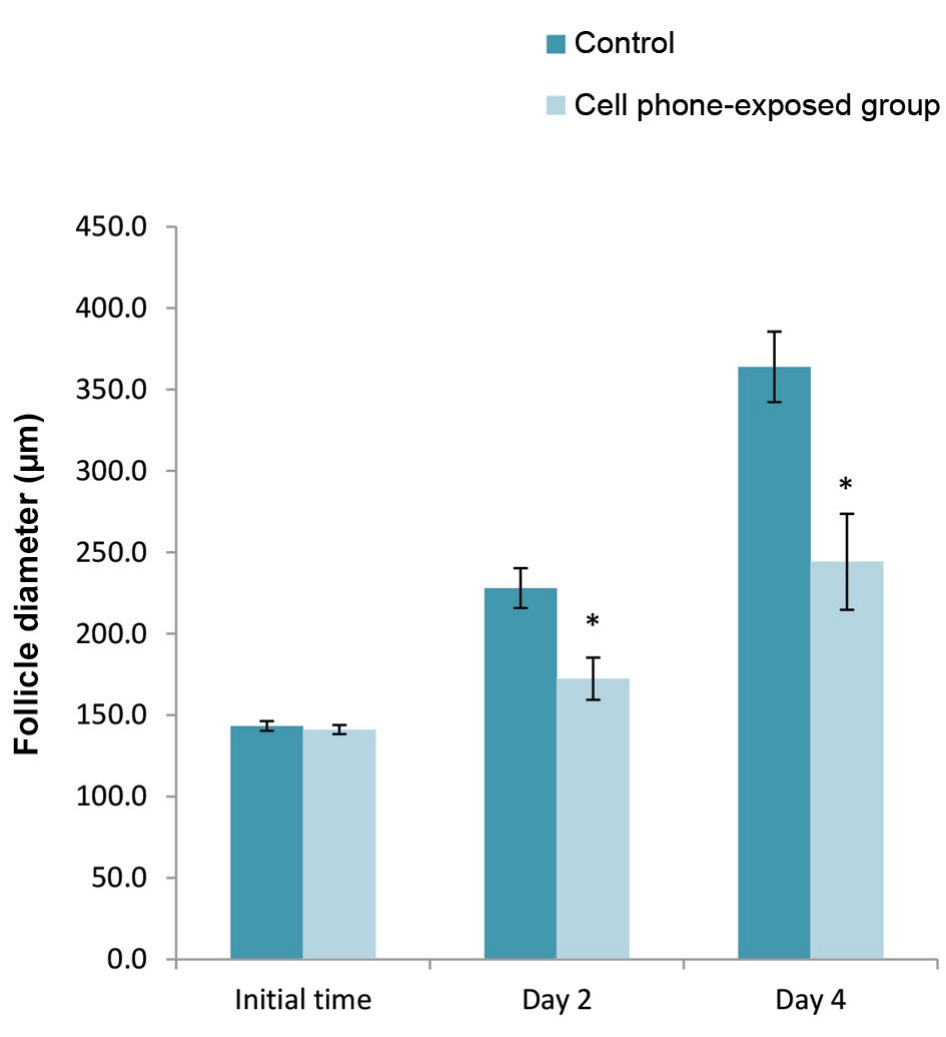
The diameter of pre-antral follicles at the initial time, days 2 and 4 of culturing. Data are expressed as the mean ± SD. *; Indicates a significant difference compared with the control (P<0.05).

Pre-antral follicles were monitored and evaluated morphologically every other day during the *in vitro* culture period. On the first day of the culture, there was no significant difference between the diameter of pre-antral follicles in the control group and the cell phone-exposed group (P>0.05, Fig .1). Whereas, at day 2 and 4 of the culture period, the diameter of the cultured pre-antral follicle in the cell phone-exposed group was significantly decreased compared with the control group (P<0.05, Fig .1). 

**Fig.2:**
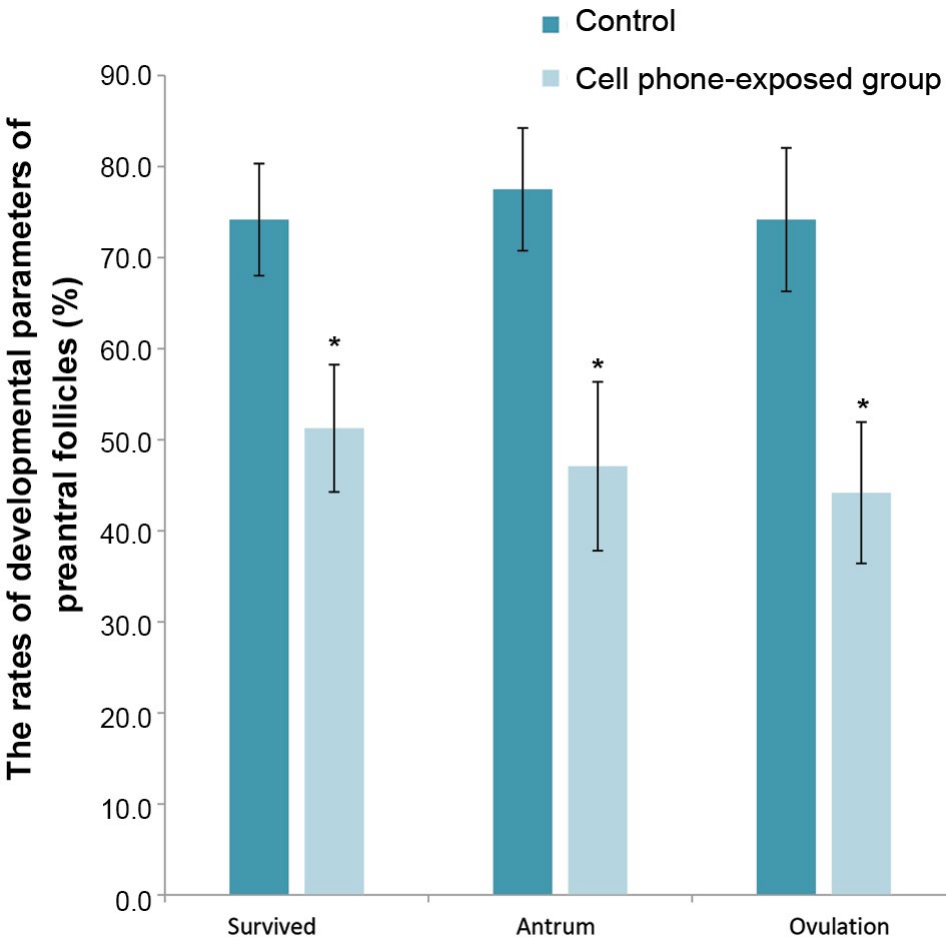
The rate of developmental parameters of pre-antral follicles. Data are expressed as the mean ± SD. *; Indicates a significant difference compared with the control (P<0.05).

**Fig.3:**
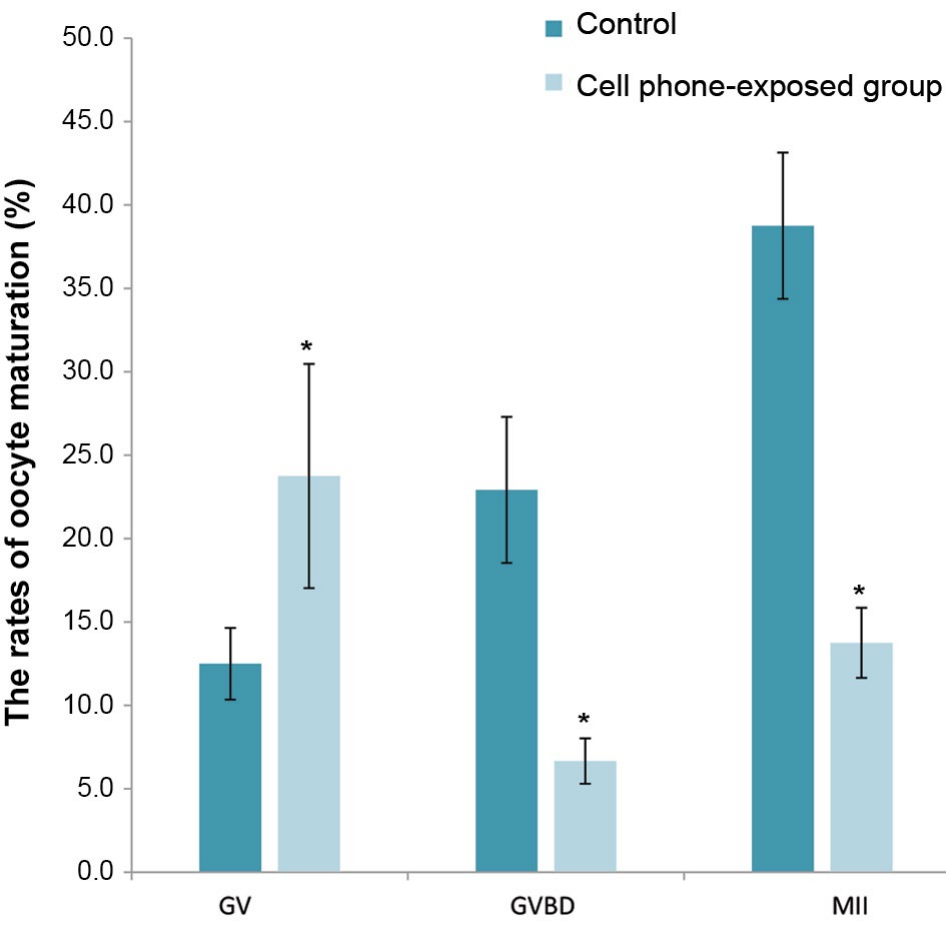
The rates of the oocyte maturation. Data are expressed as the mean ± SD. *; Indicates a significant difference compared with control (P<0.05), GV; Germinal vesicle, GVBD; Germinal vesicle breakdown, and MII; Metaphase II oocytes.

Additionally, the results revealed that the survival rate of follicles was significantly higher in the control group compared with the cell phone-exposed group (P<0.05, Fig .2). The antral formation rate of cultured pre-antral follicles in the cell phone-exposed group was significantly decreased compared with the control group (P<0.05, Fig .2). After 12 days of the culture period and induction of ovulation, the rate of ovulation in pre-antral follicle exposed to the cell phone was statistically decreased in comparison with the control group (P<0.05, Fig .2). Also, the rates of MII and GVBD oocytes were significantly lower in the cell phone-exposed group than the control group (P<0.05, Fig .3), while, the GV rate of the cell phone-exposed group was increased significantly compared with the control group (P<0.05, Fig .3). 

### Effect of electromagnetic radiation on the gelatinase activity of pre-antral follicles

**Fig.4:**
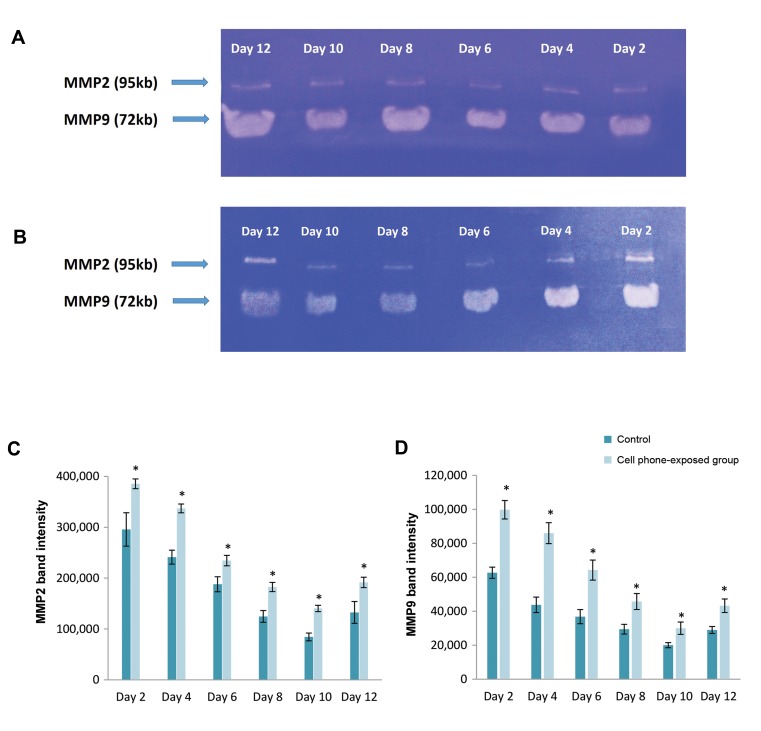
MMP-2 and-9 activities of *in vitro* cultured pre-antral follicles with or without cell phone exposure determined by zymography as described in the text. **A.** Gelatin zymography of the control group, **B.** Gelatin zymography of cell phone-exposed group, **C.** Relative optical density of MMP-2, and **D.** Relative optical density of MMP-9. Data are expressed as the mean ± SD. *; Indicates significant a difference compared with control (P<0.05).

**Fig.5:**
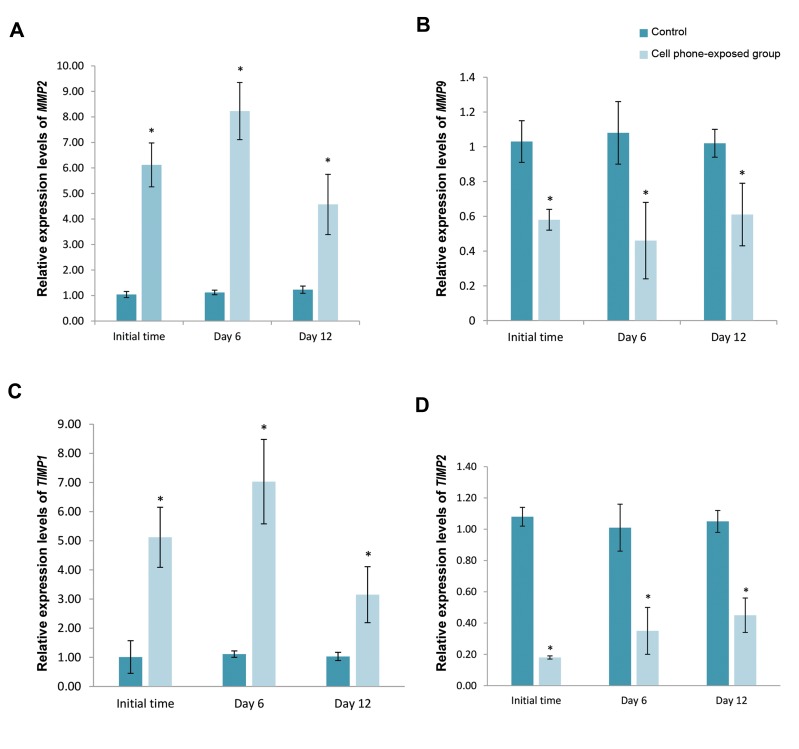
The mRNA expression levels of *MMP-2, MMP-9, TIMP-1*, and *TIMP-2* in pre-antral follicles during *in vitro* culture with or without (control) cell phone exposure. **A.** The mRNA expression levels of *MMP-2*, **B.** The mRNA expression levels of *MMP-9*, **C.** The mRNA expression levels of *TIMP1*, and *D.* The mRNA expression levels of *TIMP2*. Data are expressed as the mean ± SD. *; Indicate a significant difference compared with control (P<0.05).

The gelatinase activity of both MMP-2 and -9 was evaluated in cultured pre-antral follicles through gelatin zymography. The gelatin activity of both MMP-2 and -9 was decreased during the culture period in pre-antral follicles of the control and cell phone-exposed groups, whereas in the 12th days of the culture period, the activity was increased upon the addition of human chorion gonadotrophin (hCG) to the culture medium (P<0.05, Fig .4). Additionally, as shown in Figure 5, it was found that the *MMP-2* activity in the pre-antral follicles of the cell phone-exposed group was significantly lower in comparison with the control group (P<0.05). Similar conditions were found for the *MMP-9* activity, and thus, the activity of *MMP-9* in pre-antral follicles of the cell phone- exposed was significantly lower than the control group during culture period (P< 0.05, Fig .4). 

### Effect of electromagnetic radiation on the gene expression of *MMP-2* and *-9*, as well as *TIMP-1* and *-2*

The expression of the *MMP-2* and -9, as well as the *TIMP-1* and -2 genes are shown in Figure 5. The results show that the gene expression of the *MMP-2* gene was significantly increased in pre-antral follicles of the cell phone- exposed group compared with the control group during *in vitro* culture (P<0.05). Inversely, the gene expression of the *MMP-9* gene was markedly decreased in pre-antral follicles of the cell phone-exposed group compared with the control group during *in vitro* culture. Furthermore, the gene expression of *TIMP-1* showed a significant increase in preantral follicles of the cell phone-exposed group compared with the corresponding values in the control group during culture period (P<0.05), whereas, the *TIMP-2* expression in pre-antral follicles of the cell phone-exposed group was significantly reduced during the culture period compared with the control groups (P<0.05). 

## Discussion

To date, there are a lot of conflicting reports on the adverse effects of mobile phones on human health. These contradictory reports have been attributed to the difference in exposure time, variable frequencies, types of tissue, etc. (17-19). The mechanism of EMR, emitted by mobile phone has not been well understood. Nevertheless, it has been shown that EMR cause changes in the cell membrane integrity and activity of different enzymes (17, 18). 

In the present study, we showed that exposure to the mobile phone could damage the development of preantral follicles, decrease the number of ovulated oocytes, and increase the number of arrested GV oocytes. The regulated interplay between different cells, hormones, and various macromolecules are necessary for ovarian folliculogenesis (20). In the present study, incomplete oocyte nuclear maturation, at least to some extent, could be explained by EMR-induced apoptosis in somatic cells of pre-antral follicles, particularly the granulosa cells and reduced proliferation as shown previously (21). Although the precise mechanism of EMR is unknown, another explanation might be the inhibition of cell growth, protein misfolding, and detrimental effects on cellular signaling (18). Furthermore, in the *in vivo* condition, mobile phone radiation could induce oxidative stress (OS) via an increase in the reactive oxygen species (ROS) production and a reduction in antioxidant activity of enzymes (22). In this regard, Mao et al. (23) showed that EMR increases ROS production and decreases the activity of enzymatic antioxidants. Also, Agarwal et al. (24) indicated that cell phone radiation increases the generation of ROS and MDA, while, it can decrease the antioxidant enzyme activity in semen plasma, suggesting that exposure to the cell phone has unfavorable effects on the fertility potential of spermatozoa (6). It has also been shown that cell phone radiation induces apoptosis and OS and it is capable of reducing the total antioxidants capacity in follicular granulosa cells (25) which, in turn, led to the reduced number of ovarian pre-antral follicles (26). 

It has been also demonstrated that the induction of oxidative stress, as a result of the mobile phone exposure may damage the ECM (27). The results of the current study showed that exposure to mobile phone altered the MMPs activity and their inhibitors in cultured pre-antral follicles. The activity of MMPs in the ovarian cycle is pre-requisite for the ECM remodeling and follicular development. MMPs and TIMPs have critical roles in this process such as theca cells differentiation, proliferation/differentiation of the granulosa cells, antrum formation, the formation of the basement membrane, and ovulation (28). Thus, any change in the normal ECM remodeling and the activity of MMPs could interrupt the process of folliculogenesis. This confirms the results of the present study because the gelatinase activity and expression levels of *MMP-2* and -9 and their tissue inhibitors changed upon exposure to cell phone radiation which, in turn, led to the impairment in the development of cultured pre-antral follicles. 

Gelatinase (*MMP-2* and 9) plays a crucial role in the membrane destruction in the ovulation process, which separates granulosa and thecal layers and it can hydrolyze collagen fibers. Hence, dysregulation in gelatinase function leads to the perturbations in the folliculogenesis and ovulation processes. This finding is in agreement with our results, wherein changes in the expression of MMP2 and -9, as well as the gelatinase activity significantly reduced the follicle development and ovulation. It has been suggested that MMPs play a significant role in follicular atresia; so, increased activities of *MMP-2* and -9 in the follicular fluid is associated with the induction of atresia (10). These observations confirm the findings of the present study indicating that a higher degeneration rate in cell phone-exposed pre-antral follicles is associated with increased activity of both *MMP-2* and *-9*. The results of the present study demonstrated that the activity of *MMP-2* and *-9* is increased in day 12 of the cultivation period followed by the administration of hCG in both cell phone- exposed and untreated pre-antral follicles. This change appears to be due to the addition of hCG to the culture medium that causes the induction of ovulation. In the in vivo condition, ovulation is a dynamic process initiated with the luteinizing hormone (LH) surge, follicular wall rupture, and oocyte release (20). Pre-ovulatory LH surge is an endocrine signal for ovulation. The LH surge along with biochemical events involved in the synthesis and secretion of prostaglandins, progesterone, cytokines, and growth factors is closely related to proteolytic enzyme activities, such as MMPs. Evidence suggests that proteolytic destruction of the ECM at the apex of pre-ovulatory follicles before the ovulation process is the essential stage in the onset of the LH surge, whereas the synthesis of MMP inhibitors inhibit the ovulation process (29). Therefore, LH-induced proteolysis activity plays a vital role in the ovulation process. In this regard, the activity of gelatinase A in ovine follicles is increased followed by the LH surge (30). This situation was also found in rats (31), which was similar to the results of our study. The supplemented culture medium with hCG increased the activities of MMP-2 and -9. The activity of TIMPs was also concomitantly regulated with MMPs. Therefore, the activity of TIMPs is negatively correlated with the activity of MMPs. 

## Conclusion

The results of the present study demonstrated that cell phone radiation changes gelatinolytic activity linked to MMP-2 and -9, leading to decreased developmental competence of mouse pre-antral follicles. However, in the *in vivo* condition, ovarian tissue and cell phone are separated by several tissue layers; therefore, further studies are warranted to mimic the *in vivo* condition for cultured pre-antral follicles to evaluate the effect of cell phone radiation. 

## References

[1] Wyde ME, Horn TL, Capstick MH, Ladbury JM, Koepke G, Wilson PF (2018). Effect of cell phone radiofrequency radiation on body temperature in rodents: Pilot studies of the National Toxicology Program’s reverberation chamber exposure system. Bioelectromagnetics.

[2] Ayata A, Mollaoglu H, Yilmaz HR, Akturk O, Ozguner F, Altuntas I (2004). Oxidative stress‐mediated skin damage in an experimental mobile phone model can be prevented by melatonin. J Dermatol.

[3] Oyewopo AO, Olaniyi SK, Oyewopo CI, Jimoh AT (2017). Radiofrequency electromagnetic radiation from cell phone causes defective testicular function in male Wistar rats. Andrologia.

[4] Rao VS, Titushkin IA, Moros EG, Pickard WF, Thatte HS, Cho MR (2008). Nonthermal effects of radiofrequency-field exposure on calcium dynamics in stem cell-derived neuronal cells: elucidation of calcium pathways. Radiat Res.

[5] Remondini D, Nylund R, Reivinen J, Poulletier de Gannes F, Veyret B, Lagroye I (2006). Gene expression changes in human cells after exposure to mobile phone microwaves. Proteomics.

[6] Desai NR, Kesari KK, Agarwal A (2009). Pathophysiology of cell phone radiation: oxidative stress and carcinogenesis with focus on male reproductive system. Reprod Biol Endocrinol.

[7] Friedman J, Kraus S, Hauptman Y, Schiff Y, Seger R (2007). Mechanism of short-term ERK activation by electromagnetic fields at mobile phone frequencies. Biochem J.

[8] Sternlicht MD, Werb Z (2001). How matrix metalloproteinases regulate cell behavior. Annu Rev Cell Dev Biol.

[9] Singh D, Srivastava SK, Chaudhuri TK, Upadhyay G (2015). Multifaceted role of matrix metalloproteinases (MMPs). Front Mol Biosci.

[10] Goldman S, Shalev E (2004). MMPS and TIMPS in ovarian physiology and pathophysiology. Front Biosci.

[11] Baka S, Zourla K, Kouskouni E, Makrakis E, Demeridou S, Tzanakaki D (2010). Matrix metalloproteinases 2 and 9 and their tissue inhibitors in the follicular fluid of patients with polycystic ovaries undergoing in vitro fertilisation. In Vivo.

[12] Hatami S, Zavareh S, Salehnia M, Lashkarbolouki T, Ghorbanian MT, Karimi I (2014). The impact of alpha lipoic acid on developmental competence of mouse vitrified pre-antral follicles in comparison to those isolated from vitrified ovaries. Iran J Reprod Med.

[13] Toth M, Fridman R (2001). Assessment of gelatinases (MMP-2 and MMP- 9 by gelatin zymography. Methods Mol Med.

[14] Chomczynski P, Sacchi N (1987). Single-step method of RNA isolation by acid guanidinium thiocyanate-phenol-chloroform extraction. Anal Biochem.

[15] Bagheri F, Goudarzi I, Lashkarbolouki T, Salmani ME (2015). Melatonin prevents oxidative damage induced by maternal ethanol administration and reduces homocysteine in the cerebellum of rat pups. Behav Brain Res.

[16] Bustin SA, Benes V, Garson JA, Hellemans J, Huggett J, Kubista M (2009). The MIQE guidelines: minimum information for publication of quantitative real-time PCR experiments. Clin Chem.

[17] Merhi ZO (2012). Challenging cell phone impact on reproduction: a review. J Assist Reprod Genet.

[18] Gye MC, Park CJ (2012). Effect of electromagnetic field exposure on the reproductive system. Clin Exp Reprod Med.

[19] Suzuki S, Okutsu M, Suganuma R, Komiya H, Nakatani‐Enomoto S, Kobayashi S (2017). Influence of radiofrequency-electromagnetic waves from 3rd‐generation cellular phones on fertilization and embryo development in mice. Bioelectromagnetics.

[20] Baerwald AR, Adams GP, Pierson RA (2012). Ovarian antral folliculogenesis during the human menstrual cycle: a review. Hum Reprod Update.

[21] Cecconi S, Gualtieri G, Di Bartolomeo A, Troiani G, Cifone MG, Canipari R (2000). Evaluation of the effects of extremely low frequency electromagnetic fields on mammalian follicle development. Hum Reprod.

[22] Balci M, Devrim E, Durak I (2007). Effects of mobile phones on oxidant/ antioxidant balance in cornea and lens of rats. Curr Eye Res.

[23] Mao XW, Mekonnen T, Kennedy AR, Gridley DS (2011). Differential expression of oxidative stress and extracellular matrix remodeling genes in low- or high-dose-rate photon-irradiated skin. Radiat Res.

[24] Agarwal A, Desai NR, Makker K, Varghese A, Mouradi R, Sabanegh E (2009). Effects of radiofrequency electromagnetic waves (RF-EMW) from cellular phones on human ejaculated semen: an in vitro pilot study. Fertil Steril.

[25] Merhi ZO (2012). Challenging cell phone impact on reproduction: a review. J Assist Reprod Genet.

[26] Bakacak M, Bostancı MS, Attar R, Yıldırım ÖK, Yıldırım G, Bakacak Z (2015). The effects of electromagnetic fields on the number of ovarian primordial follicles: an experimental study. Kaohsiung J Med Sci.

[27] Alge-Priglinger CS, Kreutzer T, Obholzer K, Wolf A, Mempel M, Kernt M (2009). Oxidative Stress-Mediated Induction of MMP-1 and MMP-3 in Human RPE Cells. Invest Ophthalmol Vis Sci.

[28] Smith MF, Ricke WA, Bakke LJ, Dow MP, Smith GW (2002). Ovarian tissue remodeling: role of matrix metalloproteinases and their inhibitors. Mol Cell Endocrinol.

[29] Gottsch ML, Van Kirk EA, Murdoch WJ (2002). Role of matrix metalloproteinase 2 in the ovulatory folliculo-luteal transition of ewes. Reproduction.

[30] Gottsch ML, Van Kirk EA, Murdoch WJ (2000). Tumour necrosis factor alpha up-regulates matrix metalloproteinase-2 activity in periovulatory ovine follicles: metamorphic and endocrine implications. Reprod Fertil Dev.

[31] Robker RL, Russell DL, Espey LL, Lydon JP, O’Malley BW, Richards JS (2000). Progesterone-regulated genes in the ovulation process: ADAMTS-1 and cathepsin L proteases. Proc Natl Acad Sci USA.

